# Genetic analysis of a representative of the military nobility from the Dal’niy (Vydrin) burial ground of the Chernyakhov culture in the Kursk region

**DOI:** 10.18699/vjgb-26-96

**Published:** 2026-10

**Authors:** E.V. Rozhdestvenskikh, T.V. Andreeva, S.S. Kunizheva, A.D. Manakhov, M.S. Gasheva, О.А. Radyush, I.K. Reshetova, M.V. Dobrovolskaya, E.I. Rogaev

**Affiliations:** Sirius University of Science and Technology, Sirius Federal Territory, Krasnodar region, Russia; Sirius University of Science and Technology, Sirius Federal Territory, Krasnodar region, Russia Vavilov Institute of General Genetics of the Russian Academy of Sciences, Moscow, Russia Centre of Genetics and Genetic Technologies, Faculty of Biology, Lomonosov Moscow State University, Moscow, Russia; Sirius University of Science and Technology, Sirius Federal Territory, Krasnodar region, Russia Vavilov Institute of General Genetics of the Russian Academy of Sciences, Moscow, Russia; Sirius University of Science and Technology, Sirius Federal Territory, Krasnodar region, Russia Vavilov Institute of General Genetics of the Russian Academy of Sciences, Moscow, Russia; Sirius University of Science and Technology, Sirius Federal Territory, Krasnodar region, Russia; Institute of Archaeology of the Russian Academy of Sciences, Moscow, Russia; Institute of Archaeology of the Russian Academy of Sciences, Moscow, Russia; Institute of Archaeology of the Russian Academy of Sciences, Moscow, Russia; UMass Chan Medical School, Department of Psychiatry, Shrewsbury, USA

**Keywords:** Chernyakhov culture, late Roman period, ancient DNA, nomads, archaeogenetics, черняховская культура, позднеримское время, древняя ДНК, кочевники, археогенетика

## Abstract

During the late Roman period, the military and political conditions in Eastern Europe declined both along the frontier between the Roman Empire and Barbaricum and within those territories. The Chernyakhov culture played an important role in the dynamics of these events. According to historical and archaeological evidence, this society was formed by Germanic tribes that had moved into the region from northwestern Europe, as well as a nomadic population of the steppes. The Chernyakhov culture’s funerary traditions included the placement of weapons, most likely solely in the graves of military nobles. In this context, human remains from these burials serve as a unique resource for archaeogenetic studies, enabling insights into the genetic origins of the military aristocracy within the Chernyakhov culture. In this paper, we present the results of a whole-genome analysis of a member of the military elite from a status burial of the Chernyakhov culture at the Dal’niy (Vydrin) burial ground, located on the northern border of the Chernyakhov culture area. According to archaeological data and previous anthropological analysis, the buried individual belonged to the military nobility of the late Chernyakhov culture, which lasted from the fourth to fifth centuries AD. The whole-genome sequences of this individual were generated by large-scale parallel sequencing. The individual was shown to be male. Analysis of haplogroup markers indicated the European origin of maternal and paternal lineages. The examination of autosomal markers clearly indicated a genetic link between this individual, associated with the warrior class of the Chernyakhov culture, and members of nomadic steppe groups, specifically the Scythians, Sarmatians, and Avars. The results contribute to our understanding of the complex picture of interethnic contacts that created the basis for the formation of the polyethnic Chernyakhov culture.

## Abstract (ru)

В позднеримский период на территории Восточной Европы происходит обострение военно- политической обстановки как на границе Рима (Римской империи) и Барбарикума, так и внутри них. Значительную роль в динамике этих процессов играла черняховская культурная общность, формирование которой, согласно историко-археологическим данным, происходило при активном участии германских племен, продвинувшихся в этот регион с Северо-Запада Европы, причерноморского оседлого и отчасти степного кочевого населения. Погребальный обряд черняховской культуры предполагает крайне редкое помещение оружия, предположительно, только в могилы представителей воинской знати. В связи с чем антропологический материал из подобных погребений является ценным объектом для археогенетических исследований, позволяющим понять генетическое происхождение представителей воинской элиты черняховского общества. В данной работе мы впервые представляем результаты полногеномного анализа представителя воинской элиты из статусного захоронения черняховской культуры на могильнике Дальний (Выдрин), расположенного на северной границе ареала черняховской культуры. Совокупность археологических данных и проведенного ранее антропологического анализа указывает на то, что погребенный индивид принадлежал к воинской знати периода позднечерняховской культуры, который датируется концом IV в. н. э. С использованием масштабного параллельного секвенирования были определены полногеномные последовательности этого индивида. Показана принадлежность индивида к мужскому полу. Анализ однородительских маркеров свидетельствует о европейском происхождении материнской и отцовской линий исследованного мужчины. На основе анализа аутосомных маркеров мы впервые продемонстрировали генетическую связь этого индивида, представляющего воинское сословие черняховской культуры, с представителями кочевых степных групп: скифов, сарматов и авар. Полученные результаты вносят вклад в понимание сложной картины межэтнических контактов, лежавших в основе формирования населения полиэтничной черняховской общности.

## Introduction

During the Late Roman period (the 2nd–5th centuries AD), significant transformations occurred on the frontiers between Europe and the Great Steppe. This era witnessed large-scale migrations, military-political crises, and the formation of new cultural communities within the vast territories of Barbaricum, situated outside the borders of the Roman Empire (Heather, 2011). One of the most prominent among them was the Chernyakhov cultural-historical complex (or Sântana de Mureș–Chernyakhov complex, mid-3rd to early 5th century AD), which spanned extensive areas from the Lower Danube to the Seversky Donets and the Kursk region. Archaeological research confirms that the Chernyakhov culture emerged as a result of a complex polyethnic process involving the migration of Germanic tribes from Northwestern Europe and their interaction with the local populations inhabiting the forest and forest-steppe zones (Terpilovsky, 2000; Oblomskiy, 2002; Radyush, 2018, 2023). Anthropological data further indicate the possible existence of a genetic link between the Chernyakhov culture and populations from the steppe regions, specifically groups belonging to the Scythian–Sarmatian cultural circle (Scythians, Sarmatians, and Alans) (Rudich, 2006, 2010).

Despite the relative homogeneity of material culture, the funerary ritual practiced by the Chernyakhov society provides clear evidence of social hierarchy. Burials containing weapons are extremely rare and, according to the consensus view in archaeology, are attributed to representatives of the military aristocracy (Radyush, 2012, 2019; Radyush, Vasil’ev, 2022). Consequently, the anthropological remains recovered from such burials constitute a unique data source for investigating the genetic profile and biological origins of the Chernyakhov military elite, who played a pivotal role in the military-political processes that characterized the Migration Period

In 2021, archaeological excavations at the Chernyakhov burial site of Dal’niy (Vydrin) in the upper Sudzha River basin (the Kursk region) revealed a of a male aged 35–40 that was unique in the richness of its grave goods (Radyush, Vasil’ev, 2022; Radyush et al., 2025). The burial, dating back to the late 4th century, contained a significant collection of high-quality grave goods, including a large number of refined ceramic vessels, such as a massive black-polished vase, a jug, a glass goblet, belt buckles, and a fibula. A blade approximately 40 cm in length was recovered near the head of the deceased. The exceptional preservation of organic materials made it possible to establish that the weapon was in a wooden scabbard and was made of Damascus steel. Consistent with Chernyakhov funerary traditions, the individual was identified as a member of the military elite. This classification is further supported by anthropological analysis, which revealed a complex of physical characteristics typical of an experienced horseman who was regularly subjected to physical and military loads (Radyush et al., 2025). This burial is unparalleled among Chernyakhov sites on the left bank of the Dnieper, and its location in close proximity to the so-called “Knyazhye” burial near the village of Bolshoy Kamenets in the Kursk region further emphasises the high social status of the deceased male (Radyush, Vasil’ev, 2022).

Although historical scholarship traditionally recognizes the polyethnic nature of the Chernyakhov culture, the origin of its military elite, particularly in the peripheral the Left Bank Dnieper region, remains a subject of ongoing debate. Archaeogenetic research provides a powerful tool to test historical and archaeological hypotheses through the genetic analysis of individual human remains

Within the framework of this study, we present for the first time the results of a whole-genome analysis of a Chernyakhov military aristocrat, excavated from a grave at the Dal’niy (Vydrin) burial ground in the Bolshesoldatsky District of the Kursk region.

## Materials and methods

Ancient DNA (aDNA) was extracted at Sirius University in special laboratories designed to work with ancient materials. The molecular genetic work was conducted in accordance with previously developed methods (Gansauge, Meyer, 2013; Andreeva et al., 2022). Bone powder obtained from the tooth root of an individual from burial 12 at the Dal’niy burial ground (Vydrin) was used as an aDNA source. For this sample (hereinafter referred to as Dch08), two genomic libraries were prepared: one from native DNA and one from DNA treated with the PreCR Repair Mix (NEB) enzymes to repair postmortem modifications (Mouttham et al., 2015). Sequencing was performed using the Illumina MiSeq and Illumina NovaSeq 6000 platforms, generating single-end reads with a read length of 56 bp.

The initial data obtained from sequencing were analyzed using a bioinformatics pipeline, which included the following programs: FastQC, AdapterRemoval v2, mapDamage v2, BWA, SAMtools, BCFtools, BEDTools, and BamUtil (Li et al., 2009; Schubert et al., 2012, 2016; Jónsson et al., 2013; Picard Tools – By Broad Institute, 2019; Danecek et al., 2021). The trimmed reads were mapped to the reference sequence of the human mitochondrial genome (mtDNA, NC_012920.1) and to the human reference genome (GRCh37 assembly). The degree of contamination in the analyzed DNA was evaluated using two approaches: calculating mitochondrial genome heterozygosity via the Schmutzi program (Renaud et al., 2015) and examining X-chromosome polymorphisms using the ANGSD (Rasmussen et al., 2011). The genetic sex was determined for the studied individual using the karyo_RxRy program (Anastasiadou et al., 2024).

The mitochondrial genome sequence was reconstructed from a BAM file. The haplogroup was determined using HaploGrep 3 (Phylotree build 17), as described by S. Schönherr et al. (2023). The outcome was verified using YFull MTree 1.02 (YFull, 2025). We conducted a phylogeographic analysis using sequences from modern and ancient samples. The sequences were retrieved from the NCBI databases and selected via BLAST with 100 % query cover and 99.95 % identity. We also used the AmtDB dataset (Ehler et al., 2019). The phylogenetic tree was created using the mtPhyl program (Eltsov, Volodko, 2016), excluding highly variable sites.

The yhaplo program (Poznik et al, 2016) was used to determine the Y-chromosome haplogroup. To confirm the result, the identified single-nucleotide variants (SNVs) were crosschecked with the ISOGG Y‑DNA Haplogroup Tree 2019–2020 data (International Society…, 2024, https://isogg.org/tree/ index.html) and YFull YTree v.11.01.00 (YFull, 2025).

We generated a set of pseudohaploid genotypes for the 1240K panel for population genetic analyses (Mathieson et al., 2015). Genotype calling was performed using SAMtools mpileup and the --randomHaploid option from the pileupCaller package (Schiffels, 2025). Our dataset included individuals from the AADR (Allen Ancient DNA Resource) database dating from 6050 BC to 1326 AD. We restricted the analysis to genotypes derived from whole‑genome sequencing data. In addition, we incorporated individuals from the Chernyakhov culture and those associated with the Scythian–Sarmatian groups (Andreeva et al., 2025; Saag et al., 2025; Schütz et al., 2025). Related individuals, as well as samples with fewer than 10,000 marker genotypes, were excluded from the analysis.

To perform a principal component analysis (PCA), we combined the selected ancient samples with modern samples from the Human Origins (HO) panel, which contains approximately 600,000 genetic markers (Lazaridis et al., 2014). Transitions, variants with a low minor allele frequency (--maf 0.01), and variants with a high percentage of missing genotypes (-- geno 0.95), were excluded from the resultant file. Principal components were computed using the smartpca module in EIGENSOFT v.6.1.4 (Patterson et al., 2006). Ancient samples were then projected onto the principal component space defined by modern populations for visualization on the PCA plot.

To prepare the dataset for ADMIXTURE analysis, we supplemented it with reference populations that maximized genetic components. The transition variants, SNPs with low minor allele frequency, and those in linkage disequilibrium were pruned using the --indep-pairwise 200 5 0.5 option in PLINK v1.9 (Chang et al., 2015). To determine the optimal number of genetic components (K), we performed 10 independent ADMIXTURE runs for each K value ranging from 2 to 20, with cross-validation error estimation (--cv) enabled. The K value corresponding to the lowest cross-validation error was chosen as optimal.

## Results and discussion

DNA sequencing from bone powder and subsequent mapping against reference genomes yielded an average nuclear genome coverage of approximately 1x and an average mitochondrial DNA (mtDNA) coverage exceeding 100x (see the Table). An elevated frequency of cytosine-to-thymine (C-to-T) transitions was observed at the ends of molecules, confirming the authenticity of the ancient DNA (aDNA) (Fig. 1). The individual’s genetic sex was determined to be male (XY), with an estimated contamination level below 4 %.

**Table 1.**
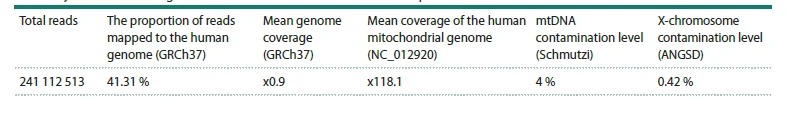
Summary statistics for the genomic data obtained from the Dch08 sample

**Fig. 1.**
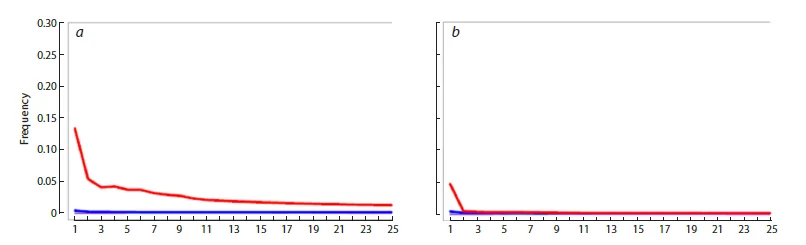
Profile of C→T substitutions (red curve) in reads mapped onto the human reference genome GRCh37. a – genomic library prepared from untreated DNA; b – genomic library prepared from repaired DNA.

The mitochondrial sequence belongs to haplogroup U5a1 and harbors additional variants at positions 12,811 and 8,610. Based on these variants, the individual clusters with a Bronze Age individual from the Late Minoan necropolis of Armenoi (Crete, Greece, 1370–1340 BC) (Lazaridis et al., 2017) as well as with modern individuals from Spain and Italy (Fig. 2). Notably, the mitochondrial genome of sample Dch08 represents a unique lineage within haplogroup U5a1, distinguished by specific variants at positions 200, 6,098, and 16,207 that are absent in both ancient and modern representatives of this clade.

**Fig. 2.**
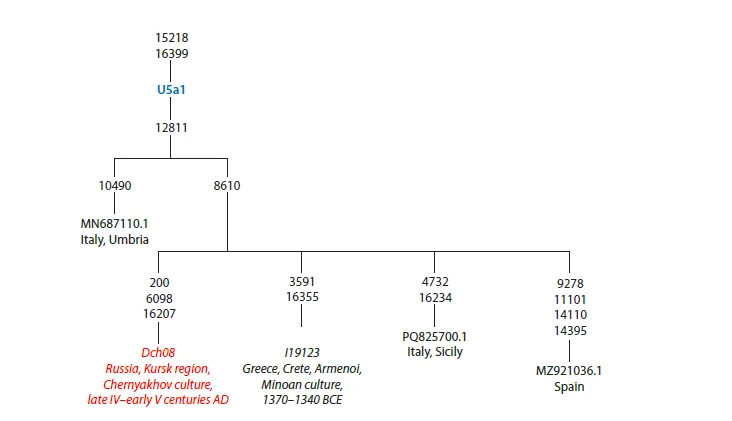
A fragment of the phylogenetic tree for haplogroup U5a1. The numbers indicate the positions of single-nucleotide substitutions. The geographical/ethnic origins of each sample are indicated, as well as the dating information for ancient samples. The test sample Dch08 is highlighted in red, and ancient samples are shown in italics.

According to YFull data, haplogroup U5a1 diverged approximately 15.4 thousand years ago and has been identified in Mesolithic populations of Northern and Eastern Europe (Skoglund et al., 2014; Mathieson et al., 2015, 2018; Günther et al., 2018; Allentoft et al., 2024), in Late Neolithic Corded Ware and Bell Beaker cultures (Linderholm et al., 2020), in Bronze Age cultures including Srubnaya (Krzewińska et al., 2018; Mathieson et al., 2018) and Minoan (Lazaridis et al., 2017), in Iron Age individuals from Italy (Emery et al., 2018 and Poland (Stolarek et al., 2023), and in Sarmatians from Kazakhstan (Damgaard et al., 2018). Currently, this lineage occurs in Italy, Spain, and Russia (in particular, in certain populations of the North Caucasus), which indicates its wide distribution in Western Eurasia.

The Y-chromosome haplotype of individual Dch08 belongs to haplogroup R1a1a1b1 (R1a-Z283). No derived markers defining downstream subclades were detected. This lineage dates to approximately 5–6 thousand years ago, and its wide distribution in Europe is associated with the expansion of bearers of the Corded Ware culture (Haak et al., 2008; Underhill et al., 2010; Saag et al., 2019). The earliest samples associated with this culture and carrying the ancestral haplogroup R1a were found at the Eulau burial ground (Eastern Germany, ~2600 BC) and in Estonia (2702 BC) (Haak et al., 2008; Saag et al., 2019). In a later period, including the Early Middle Ages, carriers of haplogroup R1a-Z283 were identified in Scandinavia and other European regions (McColl et al., 2024).

According to the FamilyTreeDNA database (https://www. familytreedna.com/public/y-dna-haplotree/R), this subclade is most widespread in modern European populations, predominantly in Poland (14.37 %), Sweden (11.62 %), and Russia (10.72 %). This result indicates the genetic continuity of the Dch08 sample along the paternal line from populations that formed in Europe during the Late Neolithic.

PCA and ADMIXTURE analyses of the Dch08 sample show a genetic profile distinct from those of previously studied Chernyakhov individuals from the Legedzine, Shyshaky, Zolochiv, and Komariv-1 sites (Järve et al., 2019; Saag et al., 2025) (Fig. 3). On the PCA plot, the individual Dch08 under study falls into the cluster of Early Iron Age nomads (Scythians and Sarmatians) and also demonstrates similarity with them according to the ADMIXTURE analysis results. Previous analyses of archaeological materials from Chernyakhov sites, as well as craniological analysis of its representatives, indicate the likely presence in this cultural community of a small fraction of individuals genetically linked to populations of the Scythian–Sarmatian groups, in the eastern and southern parts of the area (Lower Dnieper, Northern Pontic steppe regions) (Rudich, 2010, 2014; Radyush, 2023).

**Fig. 3.**
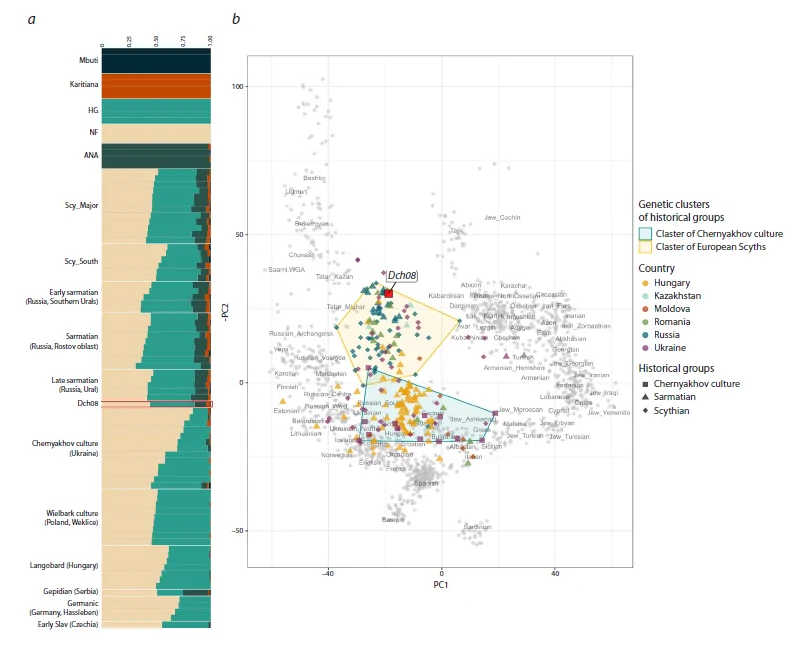
The results of ADMIXTURE (a) and PCA (b) analyses for the individual under study from the Dal’niy (Vydrin) burial ground and previously published ancient individuals Ancient group names follow the AADR database (Mallick et al., 2024) and the publications (Andreeva et al., 2025; Saag et al., 2025): NF (Neolithic Farmers) – Austria_N_LBK.SG, Turkey_Marmara_Barcin_N.SG, Germany_Saxony_LBK_N.SG; ANA (Ancient North Asian) – Mongolia_Xiongnu.SG, Russia_DevilsCave_N.SG; HG (Hunter-gatherers) – Italy_Epigravettian.SG, Russia_Mesolithic_Veretye.SG.

Our results support these hypotheses. Notably, unlike all previously published Chernyakhov culture individuals, the Dch08 individual carries Asian-related ancestry components (ANA) that are consistently present in the Early Iron Age nomadic populations. Importantly, similar genetic profiles have also been observed in Avar-period individuals from Hungary (6th–9th centuries AD) (Fig. 4), whose ancestry has been attributed to Scythian–Sarmatian sources (Maróti et al., 2022).

**Fig. 4.**
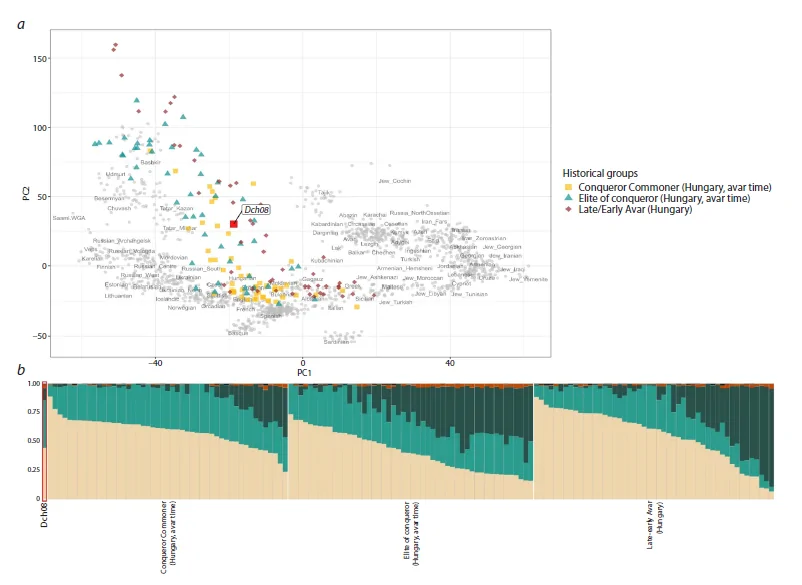
PCA (a) and ADMIXTURE (b) results for the individual from the Dal’niy (Vydrin) burial ground and previously published Avar‑period individuals from Hungary. For group designations, see Figure 3.

Historical and archaeological data suggest that migrants from Northern Europe, represented by the tribes of the Goths and the related Gepids and Heruli, as well as possibly other East Germanic groups, contributed to the formation of the Chernyakhov cultural community (Sedov, 1979; Magomedov, 2001; Shchukin, 2005). However, our analysis did not reveal any genetic similarity between Dch08 and any of these candidate groups for which whole-genome data were available at the time of this study. Specifically, no genetic link was found with individuals associated with the Wielbark culture (Stolarek et al., 2023; Antonio et al., 2024), the Lombards (Amorim et al., 2018), or the Gepids (Veeramah et al., 2018) (Fig. 3). The combination of uniparental inheritance markers of Western Eurasian origin (Y: R1a-Z283; mtDNA: U5a1) and an autosomal profile more characteristic of representatives of nomadic groups (Scythians, Sarmatians, and Avars) indicates the possible origin of individual Dch08 from a population that experienced genetic influence from nomads.

## Conclusion

Our results demonstrate the first direct genetic link between a member of the Chernyakhov warrior elite and early 1st-millennium AD steppe nomads, revealed through whole-genome sequencing of an individual from the Dal’niy (Vydrin) burial ground. The evolution of weaponry among Eastern European forest-steppe tribes, including Chernyakhov groups, was predominantly shaped by Central European and Roman cultural horizons, whereas steppe nomadic influence was initially marginal. However, during the terminal phase of the Chernya- khov culture documented at the Dal’niy (Vydrin) site, interaction with steppe groups accelerated (Radyush, 2023). This shift is explicitly supported by the steppe-derived ancestry components identified in this late-period individual’s genomic profile.

Ultimately, this study expands the empirical source base for reconstructing the mechanisms of ethnogenesis and social stratification in Eastern Europe during the pre-Hunnic and Hunnic periods, highlighting the value of integrating paleogenetic, archaeological, and historical records.

## Conflict of interest

The authors declare no conflict of interest.
